# Alliin Induces Reconstitution of Testes Damaged by Estrogen Overstimulation by Regulating Apoptosis

**DOI:** 10.3390/cimb46110776

**Published:** 2024-11-16

**Authors:** Dae-Seung Kim, Min-Jee Oh, Sang-Hwan Kim

**Affiliations:** 1 Institute of Applied Humanimal Science, Hankyong National University, 327, Jungang-ro, Unsung 17579, Gyeonggi-do, Republic of Korea; volke@naver.com; 2 General Graduate School of Animal Life Convergence Science, Hankyong National University, 327, Jungang-ro, Ansung 17579, Gyeonggi-do, Republic of Korea; wertey08@naver.com; 3 School of Animal Life Convergence Science, Hankyong National University, 327, Jungang-ro, Ansung 17579, Gyeonggi-do, Republic of Korea

**Keywords:** alliin, testes, estrogen, apoptosis, endocrine disruption

## Abstract

We analyzed the effect of alliin on the recovery of mouse testicular function and structure following estradiol treatment as well as on apoptosis regulation. During the cultivation of testicular cells, high-concentration estradiol suppressed Casp-3; PCNA, mTOR, and PI3K signaling increased; and cell proliferation in the testes was abnormally increased. Therefore, estradiol treatment increased the proportion of abnormal cells. The estradiol and 2.5 μM of alliin treatment increased Casp-3 levels and suppressed Bcl-2, PCNA, mTOR, and PI3K expression. Additionally, treatment with estradiol caused tissue loss. Furthermore, Ca^2+^ deposition decreased, TNF-r protein expression increased, and the levels of other protein markers of cell survival and death decreased. Tissue recovery and restoration of the testes occurred after alliin treatment; the gene expression of cell survival and death markers, except for TNF-r, increased with increasing Ca^2+^ deposition. Cell proliferation and tissue reorganization may correlate with an increased signal of intrinsic apoptosis owing to increased Ca^2+^ deposition. Therefore, treatment with alliin may regulate the apoptosis of cells with normal or abnormal signal transduction and help to revert testicular dysfunction.

## 1. Introduction

Hormonal imbalances can disrupt physiological and reproductive functions, particularly sexual development. Male infertility, which has been increasing globally, often results from issues related to sperm formation and production. Levine et al. [1] reported that external factors can lead to endocrine disorders and testicular dysgenesis during pubertal development. Among the various endocrine disorders that can affect males, excess estradiol secretion can cause gynecomastia during secondary sexual development and may result in growth problems due to the premature fusion of growth plates [2,3]. Elevated estradiol levels suppress the hypothalamic–pituitary–gonadal axis through negative feedback, affecting testosterone production by peripheral adipose tissue in males [4,5]. Excessive estradiol secretion contributes to male reproductive issues, including germ cell loss and impaired sperm production [3,6,7]. Exposure to estradiol in mice enhances capacitation and the acrosome reaction in sperm in vitro, whereas in vivo exposure reduces sperm survival by inducing premature capacitation [8,9]. Estradiol injections in male rats lead to seminiferous tubule cell loss, impaired sperm formation, and increased apoptosis [10]. Chronic treatment with estradiol 3-benzoate results in decreased testicular weight, spermatogenesis impairment, and increased apoptosis, attributed to elevated estrogen receptor-α expression and reduced androgen receptor expression [11,12].

Endogenous estrogen is crucial in male reproduction for regulating male fertility, prostate development, and sperm flow from the testes to the epididymis [13,14]. Although Sertoli cell tumors cause feminization, extensive studies have explored the multifaceted functions of estrogen in males. High-dose estrogen induces testicular atrophy, adipose accumulation, and Sertoli cell loss, resulting in azoospermia [6]. Estrogen-induced testicular damage may promote abnormal proliferation and Sertoli cell tumor growth, emphasizing the need for effective control strategies.

With the increasing use of natural products to control cellular remodeling by inducing apoptosis in abnormal and damaged cells, there has been a growing interest in exploring plant-derived compounds [15]. Among them, alliin, mainly derived from garlic, has antioxidant properties in relation to oxidative stress, exhibits excellent hydroxyl radical scavenging effects [16], and is known to affect the immune response of cells [17,18]. Although the exact molecular mechanisms of all compounds are not yet known, epidemiological studies have shown that the consumption of garlic, foods containing garlic, or garlic extracts reduces the risk of developing various diseases, including cancer [19,20], and is thought to affect the control of damaged cells.

We analyzed the effects of alliin, a pro-apoptotic substance, on estrogen-exposed cells to explore the effects of estrogen on cell composition. The novel functions of alliin warrant investigation, as it inhibits gastric carcinoma cell proliferation while sparing normal intestinal cells and inducing apoptosis via reactive oxygen species and key apoptotic factors [15,21]. Therefore, the aim of our study was to assess whether estrogen overstimulation in mouse testes leads to abnormal tissue damage and whether alliin can restore cellular structure and function by examining IGF/AKT signaling and apoptosis-related factors.

## 2. Materials and Methods

All animal experiments were conducted at the HanKyong National University and the safety guidelines for experimental animals were adhered to. The Institutional Animal Care and Use Committee approved the study protocol and human treatment methods (HK-2023-1).

### 2.1. Mouse Preparation

A total of 33 (ICR (Institute for Cancer Research (CD-1^®^)) 12-week-old mice with complete sexual maturity and high spermatogenesis (weight 30–35 g, male) were divided into five groups. Three mice were used for collecting testicular cells and 30 mice were used for the substance treatment. The testes were dissected using standard anatomical procedures for the cell treatment group, and the cells were harvested. The substance treatment groups consisted of 12-week-old mice housed in cages (48 cm length × 27 cm width × 20 cm height) with 6 mice per cage.

After 2 weeks of acclimation in an environment with a temperature of 20 °C and a relative humidity of 50%, the 14-week-old mice were used for the experiments. To maintain the breeding environment at a certain level, 100 g of standard feed (PURINA, St. Louis, MO, USA) and 100 mL of purified and UV-sterilized water provided by the company that provided the mice were given once every two days. After the experimental procedures, including the material treatment, were completed, the mice were euthanized using cervical dislocation.

In addition, the method of treating the mice with estrogen and administering alliin was handled according to the methods of Kaushik et al., [11] and Mansingh et al., [15,21] and efforts were made to minimize changes in the living environment such as daily mouse behavior, body weight, and water intake.

### 2.2. Testicular Cell Preparation

The cell culture and intracellular alliin treatment methods were applied with reference to methods by Park et al. [22] and Rosas-González et al. [20]. The testes of euthanized mice were dissected and minced into small pieces in 35 mm Petri dishes (Falcon, Marlboro, NY, USA). The minced testicular tissue was transferred to a 15 mL conical tube (Falcon) containing 3 mL of D-PBS. After homogenization, the mixture was centrifuged at 1500 rpm for 5 min at room temperature, and the supernatant was removed. All testes cells were cultured in 3 mL of Dulbecco’s modified Eagle’s medium (DMEM; Gibco, MA, USA) supplemented with 100 μL of an antibiotic (Gibco, Miami, NY, USA) and 5 μL of gentamicin (Sigma-Aldrich, St. Louis, MO, USA) at 37 °C in a shaking incubator for 1 h to remove contaminants. After another centrifugation was performed at 1500 rpm for 5 min at room temperature, the supernatant was discarded and the cells were counted. The cells were diluted to a density of 2 × 10^5^/mL in 10% fetal bovine serum (FBS)-containing DMEM. The prepared testicular cells were inoculated into 35 mm Petri dishes (2 mL per dish) with DMEM supplemented with 10% FBS. The medium was replaced every 48 h with fresh DMEM containing 10% FBS DMEM for one week. Non-adherent cells were removed by washing and the testicular cells were then used for analysis.

Testicular cells were divided into eight groups for treatment, as follows: control (no treatment); alliin (1.25, 2.5, and 3.75 μM); estradiol only (5 IU); and estradiol followed by alliin (1.25, 2.5, and 3.75 μM).

All groups received a single treatment at the beginning of the culture. The total number of testes cells was determined after 48 h. Cell viability was assessed using Trypan Blue staining, and morphological analysis was performed using an IX70 microscope (Olympus, Tokyo, Japan).

### 2.3. Estradiol and Alliin Injections in Mice

For excessive stimulation with estradiol (CAS Number 50-28-2, Sigma-Aldrich, St. Louis, MO, USA) [11] and treatment with alliin (CAS Number 17795-26-5; Sigma-Aldrich, St. Louis, MO, USA) [15,21], we administered double the concentration that maintained a consistent cellular response. Injections were administered in the lower abdomen (2/3 region) on both sides of the peritoneal cavity [16,23].

Estradiol was injected at a quantity of 5 IU and alliin was injected at 6.25 μM. The differences in injection schedules and treatments provided across the groups are described in Table 1. Sixteen days after the last injection, the mice were euthanized and the testes, blood, and seminal vesicle fluid were collected for subsequent analysis.

### 2.4. Enzyme-Linked Immunosorbent Assay (ELISA)

We analyzed the expression patterns of specific proteins extracted from the testicular tissue of mice using ELISA. We first coated 96-well plates with the primary antibody, anti-PCNA (sc-7907, Santa Cruz Biotechnology, Dallas, TX, USA), and incubated it at 4 °C for 24 h. After two washes with washing buffer (1× PBS + 2.5% Triton X-100), we blocked the wells with 1% skimmed milk blocking solution at 4 °C for 24 h. After additional washes, antigen–antibody reactions were induced using an horseradish peroxidase (HRP)-conjugated anti-mouse secondary antibody (ab6741, Abcam, Cambridge, UK) and reacted with a substrate solution (R&D Systems, MN, USA). The reaction was stopped using 1 M NH_2_SO_4_, and the absorbance was measured at 450 nm.

### 2.5. Immunofluorescence (IF)

The testicular cells were fixed in 4% formaldehyde for 30 min, washed with 0.2% Triton X-100 in TBS (T-TBS) and incubated overnight in T-TBS. The testicular tissue slides were deparaffinized and hydrated. Subsequently, the primary antibodies, anti-mTOR (sc-517464, Santa Cruz Biotechnology, Dallas, TX, USA), anti-Casp-3 (sc-373730, Santa Cruz Biotechnology, Dallas, TX, USA), and anti-Bcl-2 (sc-492, Santa Cruz Biotechnology, Dallas, TX, USA), were diluted to 1:250 in a blocking solution and incubated at 4 °C for 18 h. Secondary antibodies, Alexa 594 (35560; Invitrogen, Waltham, MA, USA), and Alexa 488 (A32731; Invitrogen, Waltham, MA, USA), were used, followed by counterstaining with Hoechst 33342 (Sigma-Aldrich, St. Louis, MO, USA) and mounting with H-1000 (Vector Laboratories, Newark, CA, USA) [17,24].

### 2.6. Hematoxylin and Eosin Staining

The testicular tissue slides were deparaffinized and hydrated. Deparaffinization and hydration involved xylene treatment (10 min, twice) to remove paraffin, followed by 100% and 95% ethanol treatments (10 min each) to remove xylene. The slides were stained with hematoxylin (Fisher Scientific, Waltham, MA, USA) for 3 min, washed with distilled water, and stained with eosin (Fisher Scientific) for 30 s. After further washing, the slides were dehydrated with ethanol and xylene and mounted with Permount (Thermo Fisher Scientific, Waltham, MA, USA).

### 2.7. Alcian Blue Staining

For Alcian blue staining, we diluted 100 μL of Alcian blue (Sigma-Aldrich, St. Louis, MO, USA) in 500 μL of TBS and applied it to the glass slide. The slides were then dried overnight at 37 °C in a slide warmer. After rinsing with 1× PBS, the cells were stained with Alcian blue for 1 min, followed by another rinse with 1× PBS for analysis.

### 2.8. Alizarin Red Staining

We used Alizarin Red S (ARS) (Sigma-Aldrich, St. Louis, MO, USA) on tissue sections from each treatment group to detect Ca^2+^ (calcium) deposition. The tissues were deparaffinized and hydrated to expose antigens. Subsequently, the tissues were incubated with 0.5% ARS (*w*/*v* in water; pH 6.36–6.4) for approximately 30 min at room temperature. After stopping the staining with distilled water, the tissues were dehydrated with ethanol and fixed with Permount. Ca^2+^ deposition (orange and red spots) was visualized with an AX70 microscope (Olympus, Tokyo, Japan). NIS-Elements C software (ver. 3.2) was used for the tissue analysis.

### 2.9. Real-Time PCR Analysis

The total RNA was extracted from the tissues of the mouse testis sections of each group, pulverized using TRIzol reagent (Cat No. 10296028; Invitrogen, Waltham, MA, USA), treated with DNAse (Ambion, Austin, TX, USA) according to the manufacturer’s instructions, and quantified by ultraviolet (UV) spectroscopy. First-strand cDNA was synthesized by reverse transcription of mRNA using oligo(dT) primer and SuperScript II reverse transcriptase (Cat No. 18064022; Invitrogen, Waltham, MA, USA) from 10 μg of total RNA; 100 ng of cDNA was used for real-time PCR.

Real-time PCR was performed using the One-Step SYBR RT-PCR Kit (TaKaRa, Kyoto, Japan) according to the manufacturer’s instructions. The PCR reaction included an initial step at 94 °C for 10 min, followed by 40 cycles of denaturation at 94 °C for 30 s, annealing at 55–60 °C for 30 s, and extension at 72 °C for 55 s. The final extension step was performed at 72 °C for 5 min. The program provided by QuantStudio™ 3 Real-Time PCR (Invitrogen, Waltham, MA, USA) instrument was used to analyze the cycle threshold (Ct) and obtain a semi-log amplification plot. The relative expression levels of each gene were then calculated using the 2 method and normalized to glyceraldehyde-3-phosphate dehydrogenase (GAPDH) mRNA levels (Table 2).

### 2.10. Western Blotting Analysis

Protein extracts (30 µg) from the mouse testes tissues were separated into duplicates using 13% sodium dodecyl sulfate–polyacrylamide gel electrophoresis and transferred to a polyvinylidene fluoride membrane using a semi-dry electroblotting apparatus. The membrane was blocked with 5% skimmed milk at room temperature for 2 h and washed with TBS-T (0.1% *v*/*v* Tween20, 50 mM Tris–HCl [pH 7.6], and 200 mM NaCl). Primary antibodies, including anti-ß-actin (sc-69879; Santa Cruz Biotechnology, Dallas, TX, USA), anti-mTOR (sc-517464, Santa Cruz Biotechnology, Dallas, TX, USA), anti-Casp-3 (sc-373730, Santa Cruz Biotechnology, Dallas, TX, USA), Bcl-2 (sc-492, Santa Cruz Biotechnology, Dallas, TX, USA), PI3K (sc-365290, Santa Cruz Biotechnology, Dallas, TX, USA), anti-AKT (sc-5298, Santa Cruz Biotechnology, Dallas, TX, USA), anti-TNF-r (sc-31349, Santa Cruz Biotechnology, Dallas, TX, USA), and anti-VEGF (PA5-16754, Invitrogen, Waltham, MA, USA), were used for the antigen–antibody reactions and incubated at 4°C overnight. After washing with TBS-T, membranes were incubated with secondary antibodies (HRP-conjugated anti-rabbit, anti-mouse, or anti-goat; Santa Cruz Biotechnology, Dallas, TX, USA) at room temperature for 2 h. The membrane was washed with TBS-T and developed using the Lumi-Light substrate solution. The exposed membranes were visualized using an X-ray film.

### 2.11. Immunohistochemistry (IHC)

The testes tissue slides were deparaffinized and hydrated, with the deparaffinization and hydration processes involving the removal of paraffin in xylene (twice for 10 min each), 100% and 95% EtOH (twice for 10 min each) to remove xylene, and then treated with distilled water (twice for 5 min each) to complete the hydration process. The prepared testicular tissue slides were placed in 10 mM sodium citrate, heated for 10 min to retrieve the antigen, and cooled for 20 min to reach room temperature. Endogenous peroxidases were inactivated with methanol and 3% H_2_O_2_ for 5 min and the slides were washed thrice in 1× PBS for 5 min each. The processed slides were blocked with 5% NHS and 1% normal goat serum in 1× PBS for 1 h and the blocking solution was removed. The primary antibody, anti-VEGF (PA5-16754, Invitrogen, Waltham, MA, USA), was diluted to 1:200 in blocking solution and incubated overnight at 4 °C to induce the antigen–antibody reaction. The slides were washed with 1× PBS for 5 min; the secondary antibodies HRP-conjugated anti-rabbit and anti-mouse were diluted to 1:200 in the blocking solution and incubated for 1 h at 4 °C. The slides were then washed with 1× PBS three times for 5 min each. Subsequently, the slides were reacted with ABC reagent (Vector laboratories, Mowry Ave Newark, CA, USA) for 30 min, washed three times with 1× PBS for 5 min each, and reacted with 300 µL of DAB (Vector laboratories, Mowry Ave Newark, CA, USA) for 1 to 10 min. Color development was stopped with deionized distilled water for 5 min, counterstained with acid–Schiff (PAS) reagent (Sigma-Aldrich, St. Louis, MO, USA) and hematoxylin + 4% acetic acid, and then dehydrated by treating twice with 95% and 100% EtOH, and xylene for 10 min each. The stained slides were mounted using Permount and observed [18,25].

### 2.12. Statistical Analysis

Data were analyzed for statistical significance using Welch’s *t*-test, fold change, and GLM (generalized linear model); the Statistical Analysis System version 9.4 (SAS Institute Inc., Cary, NC, USA) was used. The differences in the means of each group were analyzed using the Tukey test as a post hoc test following ANOVA; differences between groups were considered significant at *p* < 0.05. All experiments were repeated at least three times.

## 3. Results

### 3.1. Analysis of Cell Survival and Morphological Changes After the Addition of Alliin and Estrogen to Testicular Cells

**Figure 1:**
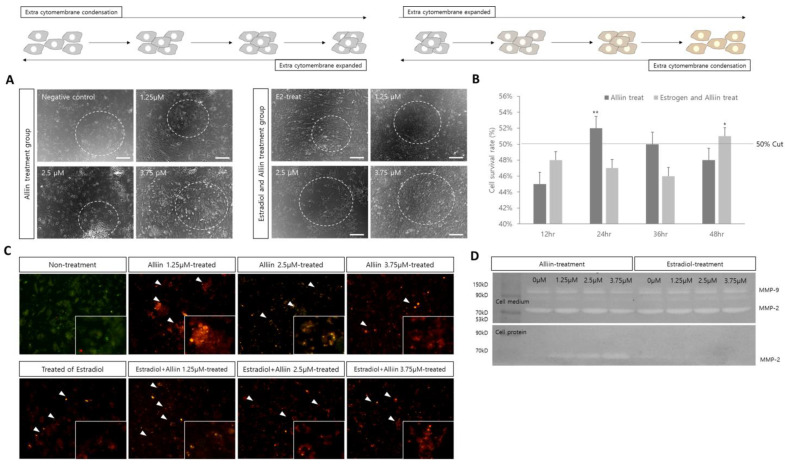
Analysis of cell survival and morphological changes in testicular cells treated with estrogen and alliin. The upper figure shows the state of testicular cells when substances were added: (**A**) morphological changes after each substance treatment in testicular cells were analyzed for cell aggregation and differentiation using a phase contrast microscope; (**B**) the number of unstained live cells was counted using Trypan Blue staining and the experiment was repeated more than three times. The average was calculated after measuring more than three times; (**C**) the expression patterns of Casp-3 and BCL-2 in cells were analyzed using immunofluorescence. Green fluorescence indicates BCL-2 expression, and red fluorescence indicates Casp-3 expression. White arrows indicate representative expression sites. All pictures are the results of Marge, and the small images are 400× magnifications of the areas where expression was prominent. The magnification of all other images is 200×; and (**D**) in situ zymography is used to analyze the activity of MMP in the cell culture medium and proteins extracted from cells. All experiments on cell survival and apoptosis factor expression patterns were repeated at least three times. *, ** the highlighted letters indicate significant differences (*p* < 0.05 and *p* < 0.01).

Figure 1 shows the results of evaluating whether alliin treatment can damage testicular cells and the effects of estrogen exposure on cell survival and cell morphology.

In normal testicular cells, cell mass increased, and clustering occurred as the alliin concentration increased. However, in the case of excessive estrogen action, condensation between the cells was controlled, the range of the cytoplasmic lumen between cells expanded, and the connection between cells was controlled (Figure 1A). When 2.5 μM of alliin was added, and cell survival was measured for up to 48 h, the cell survival rate decreased as time increased in the group treated with excessive estrogen (Figure 1B). Similar results were observed for apoptotic factors. When 1.25 to 3.75 μM of alliin was added to normal cells, Casp-3 expression was dominant; however, there was some BCL-2 expression. The 2.5 μM alliin treatment group that affected cell viability had high BCL-2 expression levels. However, in the estrogen-treated group, Casp-3 expression and apoptosis increased as alliin concentration increased (Figure 1C). Analysis of the cell matrix restructuring action based on the activity level of MMP revealed that all treatment groups showed similar activity in the cell culture medium. However, as the concentration of alliin increased in the normal cell group, the activity of MMP-2 increased (Figure 1D).

### 3.2. Analysis of Morphological Differences in Mouse Testes Treated with Estrogen and Alliin

**Figure 2:**
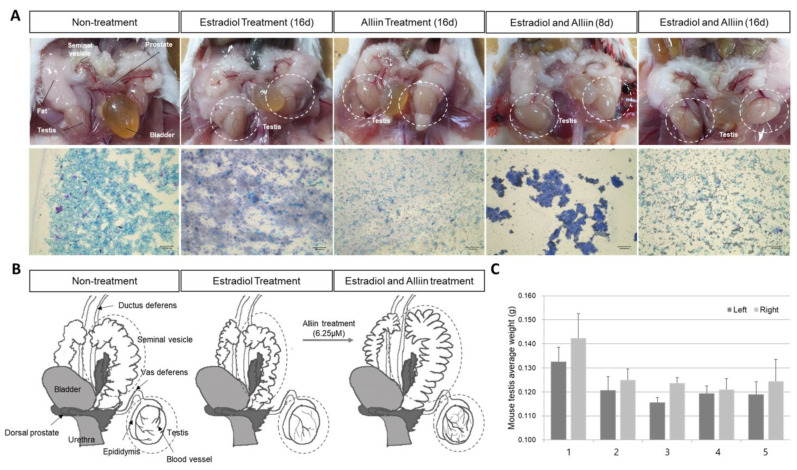
Anatomical changes and analysis of the properties of seminal vesicle fluid after estrogen overstimulation in male mice treated with alliin: (**A**) anatomical comparison of the testes of each treatment group and analysis of the properties of seminal vesicle fluid; (**B**) morphological changes in the anatomy of the genitalia after dissection; and (**C**) the thickness of the dissected testes according to each treatment group. Five animals were dissected for comparison in each group, and the dissection photos and seminal vesicle fluid analysis results are presented in randomly selected representative photos (1, 2, 3, 4, and 5 are the same as the labels of the material treatment groups shown in Table 1). The mouse model experiment involved a total of six mice per treatment group; representative models were randomly selected to be used as the result photographs.

Compared with the untreated group, the estrogen- and alliin-treated groups showed prominent changes in the size of the testes and seminal vesicle (Figure 2). In addition, there was a difference in the distribution of mucopolysaccharides in semen. When the normal group was treated with alliin for 16 days, the semen showed some viscosity, and the size of the testes decreased. In the group that was continuously overstimulated with estrogen for 8 and 16 days, semen clumping increased, and the viscosity and size of the seminal vesicles decreased. The changes in seminal vesicle size and testes weight were smaller in the estrogen-treated group than in the normal group. When overstimulation with estrogen continued for 8 or 16 days, and alliin was simultaneously administered for 8 or 16 days, the morphology of the testes and the properties of the semen in the group treated with alliin for 16 days were similar to those in the normal group. As shown in Figure 2, when there was excessive estrogen stimulation, blood vessel formation in the testes decreased, and the size and shape of the seminal vesicle changed. In contrast, the group treated with alliin showed a similar shape of the testes and seminal vesicle to those of the normal group, and blood vessel formation in the testes also significantly expanded (Figure 2B).

### 3.3. Changes and Restructuring of Testicular Tissue Owing to Excessive Stimulation by Hormones

**Figure 3:**
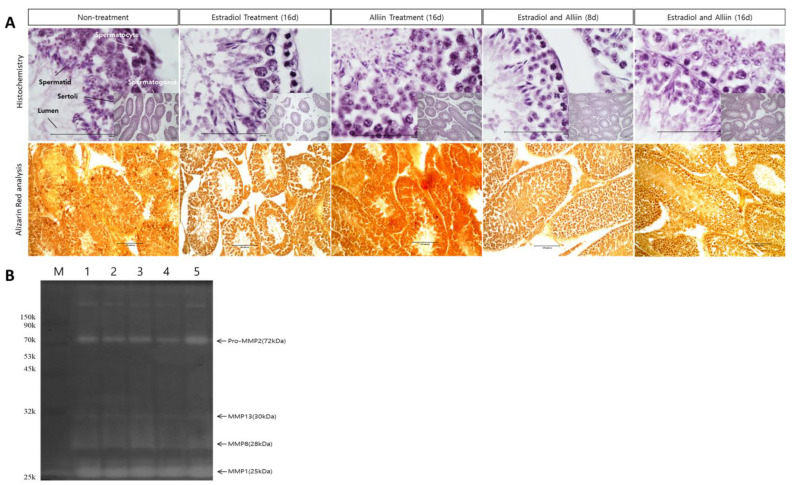
Morphological differences in testes tissue and measurement of MMP activity: (**A**) the testes of each treatment group were analyzed using hematoxylin and eosin (H and E) staining (top pictures), and Ca^2+^ response was measured (bottom pictures). The large image of the H and E staining is at 400× magnification, and the small image and Ca^2+^ analysis picture are at 100× magnification; and (**B**) in situ zymography to analyze the activity of MMP in proteins extracted from the testes (1, 2, 3, 4, and 5 are the same as the labels of the material treatment groups shown in Table 1). For histological analysis, experiments were conducted on the testes tissues of mice from all treatment groups, and all experiments were performed at least three times; the most average results are reported.

Analysis of the changes in the testicular tissue of each treatment group revealed that, after excessive stimulation with estradiol only, the lumen in the seminiferous tubules and the space between the seminiferous tubules increased (Figure 3). After alliin treatment, the lumen formed a shape similar to that of normal tissue, and seminiferous tubule development increased. In particular, when alliin was administered for 8 days after estradiol stimulation, the average size of the testicular tissue significantly increased; when alliin was administered for 16 days, the seminiferous tubules developed the most. Cell growth and development also increased, compared with those in the seminiferous tubules of the normal group when normal tissues were treated with alliin (Figure 3A). All groups treated with alliin showed high deposition in the Ca^2+^ response analysis, and MMP activity increased compared with that in the other treatment groups when alliin was simultaneously administered with estradiol for 16 days (Figure 3B).

### 3.4. Changes in IGF/mTOR Signaling After Alliin Treatment

**Figure 4:**
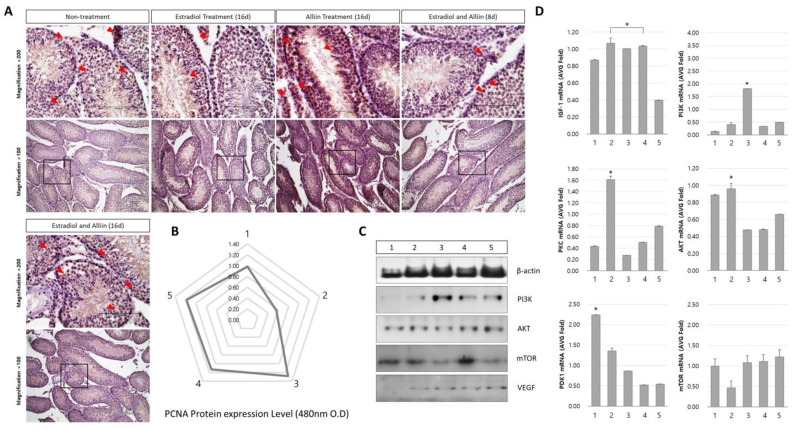
Analysis of IGF/mTOR signal and VEGF expression patterns in the testes: (**A**) protein expression and localization were analyzed using immunohistochemistry; (**B**) the expression pattern of PCNA, a cell survival factor, was determined using enzyme-linked immunosorbent assays; (**C**) the expression pattern of specific genes in the testes protein was determined using Western blotting, and quantitative analysis was performed using ß-actin; and (**D**) the expression patterns of major mRNAs belonging to the IGF/mTOR signal were determined using real-time PCR (1, 2, 3, 4, and 5 are the same as the labels of the material treatment groups shown in Table 1). Red arrows indicate representative gene expression sites. For statistical results of all experiments, real-time analyses, including IHC and Western blot, were performed at least three times for each mouse gene in each group. * The highlighted letters indicate significant differences (*p* < 0.05).

When comparing the changes in IGF signaling and the patterns of angiogenic factors, there was a large difference between the estradiol overstimulation and alliin mixed treatment groups. The angiogenic factor VEGF was expressed in all treatment groups (Figure 4A). However, it was generally highly expressed in the seminiferous tubules of the normal group treated with alliin and the group treated with estradiol overstimulation and alliin for 16 days. The expression of PCNA significantly increased in all treatment groups with alliin (Figure 4B), especially in the group treated with estradiol and alliin for 8 days. The expression patterns of IGF/mTOR and VGEF proteins were generally lower in the group treated with only estradiol than in the group treated with alliin. However, in the group treated with alliin, the expression of all proteins (PI3K, AKT, mTOR, and VEGF) increased; the expression pattern generally increased when alliin was administered for 8 to 16 days compared with that in other treatment groups (Figure 4C).

The mRNA expression pattern was different from that of the protein. Unlike the results described thus far, IGF-1, PKC, and AKT were significantly expressed in the group treated with estradiol for 16 days (Figure 4D).

### 3.5. Effect of Alliin Treatment on the Expression of Key Apoptosis Factors

**Figure 5:**
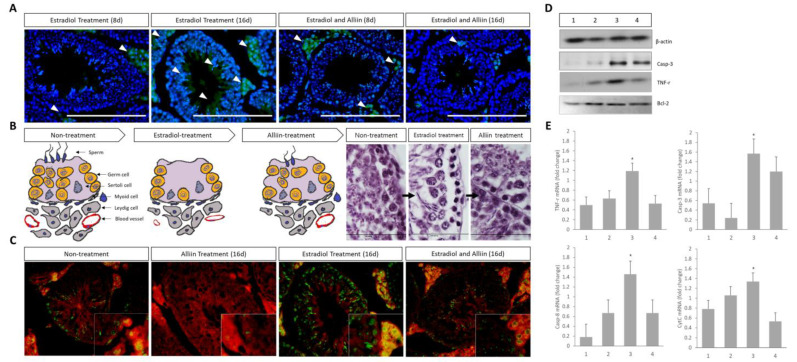
Analysis of the expression patterns of major apoptosis-related factors according to each treatment group: (**A**) analysis by immunofluorescence to compare the expression patterns of Casp-3 in testicular tissue. Blue is the nucleus of the cell stained with DAPI, and green is the expression pattern of Casp-3. White arrows indicate representative expression sites; (**B**) based on the apoptosis results, the changes in major cells of testicular tissue are graphically displayed and verified by H and E analysis results (400× magnification); (**C**) the expression sites and patterns of mTOR and Casp-3 in testicular tissue were determined. The large and small images are at 200× and 400× magnifications, respectively. Green and red represent the expression of Casp-3 protein and mTOR, respectively; (**D**) the expression pattern of specific genes in the testes protein was determined using Western blotting, and quantitative analysis was conducted using ß-actin; and (**E**) the expression pattern of the major apoptosis marker mRNA was determined using real-time PCR. The following items shown in the Western blotting and real-time PCR analyses indicate the treatment groups shown in (**D**): 1 (Non-Treatment), 2 (Alliin Treatment Day 16), 3 (Estradiol Treatment Day 16), and 4 (Estradiol and Alliin Day 1). To accurately determine the gene expression spots of immunofluorescence, the analysis was performed at least three times. To analyze the statistical significance of the gene expression patterns, all experiments were performed at least three times and analyzed. * Highlighted letters indicate significant differences (*p* < 0.05).

The results of the analysis as to whether alliin treatment affects estradiol overstimulation are shown in Figure 5. To identify the expression spots of the Casp-3 gene, an apoptosis marker, according to the location of cells in the tissue, immunofluorescence analysis was performed. As estradiol stimulation increased from day 8 to day 16, Casp-3 expression was centrally expressed in the seminiferous tubules. In contrast, when cells were treated with alliin from day 8 to day 16, Casp-3 expression decreased but was partially expressed in Leydig cell sections. Therefore, adding alliin increased cell proliferation in sections damaged by estradiol (Figure 5A,B). The expression of Casp-3 and mTOR in the untreated group was determined. Casp-3 expression was high in the group treated with estradiol overstimulation and mTOR expression increased after alliin treatment. When alliin was administered for 16 days in the estradiol-overstimulated group, Casp-3 expression in the Leydig cell zone decreased and mTOR expression increased (Figure 5C). Similar protein expression patterns were observed. In the untreated and alliin-only groups, Casp-3 expression was relatively lower than that in the estradiol treatment group; however, the expression of TNF-a showed a slight difference. That is, when alliin was added to the untreated and estradiol overstimulation groups, the expression of TNF-a increased compared to that in the other treatment groups. BCL-2, which controls the activity of Casp-3, was expressed in all treatment groups; however, the estradiol overstimulation group showed lower BCL-2 expression than the other treatment groups (Figure 5D). The mRNA expression pattern was similar to that of protein. In all results, the expression of apoptotic factors was significantly lower in the alliin treatment group (Figure 5E).

## 4. Discussion

Estradiol in men and women has a similar structure and is produced by the conversion of testosterone by aromatase [19,26]. The testes, a male reproductive organ, regulates various factors related to spermatogenesis, including germ cell proliferation, differentiation, survival, and apoptosis [27,28,29]. In addition, it plays an important role in cell proliferation, ion transport regulation, and apoptosis regulation in Sertoli cells [30,31,32]. It inhibits testosterone production and the regeneration of Leydig cells [14,33], and affects sperm concentration, motility, and morphology by being involved in the reabsorption of body fluids in the efferent ductulus [13]. Therefore, estrogen plays a role in regulating sperm production and negative feedback in males. However, excessive estrogen stimulation causes the reduced weight of reproductive organs, spermatogenesis disorder, and increased apoptosis of germ cells, which, as in our study results, shows differences in blood vessel formation within the testes and results in morphological changes in the seminal vesicles [6,8]. This can be observed as a decrease in androgen receptor expression owing to an increase in the expression of estrogen receptor-α [3,11]. The hypersecretion of estradiol causes the collapse of the cell matrix in several areas, except for the spermatogonia area in the testes, and seems to impair the function of the testes by aggravating the collapse of the spermatocytes, spermatids, and Sertoli cell areas [6,10,12,23,34].

Alliin is known to affect the reorganization of normal cells and abnormal cells and to induce appropriate apoptotic factors [20] that result in low damage to normal cells and apoptosis in abnormal cells. This suggests that alliin treatment can have a positive effect on cell activity [16,19]. In other words, alliin can affect the activity of normal cells in addition to controlling cancer growth.

In the present study, the analysis of Ca^2+^ activity and apoptosis showed that estradiol treatment decreased the Ca^2+^ deposition rate. However, alliin treatment had the opposite effect, increasing the Ca^2+^ deposition rate. Therefore, the increase in Ca^2+^ deposition by alliin treatment may induce intrinsic apoptosis owing to endoplasmic reticulum (ER) stress and may regulate apoptosis by reducing external cell death [17,35]. Considering that alliin treatment increased the activity of cell survival factors centered on the intracellular matrix, such as mTOR, PI3K, VEGF, AKT, and PCNA, tissue restructuring is thought to be achieved by appropriately removing abnormal cells in the testes tissue using apoptosis. Alliin increases the deposition rate of Ca^2+^ in testicular tissue damaged by estrogen overstimulation, aggravating ER stress, and may increase the activity of intrinsic apoptosis by activating the apoptotic signal by reactive oxygen species in the mitochondria [36,37,38]. Although the exact reason for this is unknown, alliin treatment appeared to affect cell development by increasing intracellular IGF signaling, activating the mTOR mechanism to increase protein synthesis in the ER. These results suggested that estradiol administration decreased the diameter of seminiferous tubules, fatty acid degeneration and accumulation in the testes tissue, and the loss of Sertoli cells in the seminiferous tubules in mice [6,10,11]. Estradiol is thought to regulate apoptosis in cells that perform abnormal or normal signaling by acting on abnormal tissue composition where glycoprotein synthesis and nuclear and cytoplasmic degeneration occur in Leydig cells, which may play an important role in tissue restructuring [6,29,34]. Therefore, the results of the present study suggest that alliin can restore damaged testicular tissue to its normal form. Alliin treatment is suggested to increase TNF-ɑ/Casp-3 signals in damaged Leydig cells and seminiferous tubules when abnormal hormonal action is excessive and activate IGF/mTOR signals in some cells that perform normal signaling, thereby inducing tissue reconstruction.

We evaluated whether Alliin treatment could affect cell reorganization in damaged testes in animal clinical trials and obtained results indicating that Alliin administration could induce the reorganization of damaged cells. However, continued research is needed to determine whether the values used in animal clinical trials can be applied to human subjects. However, at least through this study, we believe that the appropriate use of Alliin can positively affect damaged testes.

## 5. Conclusions

This study aimed to analyze the action of alliin, which can be extracted from garlic, as a substance that can restore testes function. Excessive estrogen stimulation causes functional and morphological alterations in the testes that maximize its apoptotic action. Accordingly, we showed that appropriate treatment with alliin restored testes’ function by inducing IGF/mTOR signaling and regulating the expression of Casp-3 to activate the reconstructive action of the main cells of the damaged testes. Alliin affects the functional loss of the testes and the process of restoring it by regulating cell activity in tissues damaged by estrogen, providing important research data for the treatment of male infertility in the future.

## Figures and Tables

**Table 1:** Estradiol and alliin treatment in mice.

Group	Compound	Schedule
1	None	Saline solution (50 μL) was injected for 16 days.
2	Estradiol	Estradiol (5 IU/50 μL) was injected for 16 days.
3	Alliin	Alliin (6.25 μM/50 μL) was injected for 16 days.
4	Estradiol and alliin	Estradiol (5 IU/50 μL) was injected for 16 days. Estradiol was injected for 8 days, and then estradiol (5 IU/50 μL) and aliin (6.25 μM/50 μL) were injected for 8 days.
5	Estradiol and alliin	Estradiol (5 IU/50 μL) and alliin 6.25 μM were injected for 16 days.

**Table 2:** Oligonucleotide primers used to amplify target genes for real-time PCR analysis.

Target Gene	NCBI Gene ID	Primer Sequence (5′–3′)		Tm (°C)
*Mos-Gapdh*	NM_001289726.2	Forward Reverse	CCCGTTCGACAGACAGCCGTG CCGCCTTGACTGTGCCGTGG	56
*Mos-IGF-1*	NM_001111274.1	Forward Reverse	TGTCGTCTTCACATCTCTTCTACCTG CCACACACGAACTGAAGAGCGT	60
*Mos-PI3K*	NM_001024955.2	Forward Reverse	AGGAGCGGTACAGCAAAGAA GCCGAACACCTTTTTGAGTC	55
*Mos-PDK1*	NM_001360002.1	Forward Reverse	CCGGGCCAGGTGGACTTC GCAATCTTGTCGCAGAAACATAAA	60
*Mos-PKC*	NM_011101.3	Forward Reverse	TGGGGTCCTGCTGTATGAGA TCAAAGTTTTCGCCACTGCG	55
*Mos-AKT*	NM_001165894.2	Forward Reverse	TGAAAACCTTCTGTGGGACC TGGTCCTGGTTGTAGAAGGG	55
*Mos-mTOR*	NM_020009.2	Forward Reverse	CTGGGACTCAAATGTGTGCAGTTC GAACAATAGGGTGAATGATCCGGG	58
*Mos-TNF- a*	NM_001278601.1	Forward Reverse	CCACCACGCTCTTCTGTCTAC AGGGTCTGGGCCATAGAACT	58
*Mos-Casp-3*	NM_001284409.1	Forward Reverse	CCTCAGAGAGACATTCATGG GCAGTAGTCGCCTCTGAAGA	55
*Mos-Casp-8*	NM_001080126.2	Forward Reverse	GCAGAAAGTCTGCCTCATCC GGCCTCCATCTATGACCTGA	55
*Mos-CytC*	NM_025710.2	Forward Reverse	GAGGCAAGCATAAGACTGGA TACTCCATCAGGGTATCCTC	52

## Data Availability

The original contributions presented in the study are included in the article, further inquiries can be directed to the corresponding authors.
